# Implementation of maternal and perinatal death surveillance and response system among health facilities in Morogoro Region: a descriptive cross-sectional study

**DOI:** 10.1186/s12913-021-07268-5

**Published:** 2021-11-17

**Authors:** Christina Jacob Kashililika, Fabiola Vincent Moshi

**Affiliations:** 1 Department of Clinical Nursing School of Nursing and Public Health, The University of Dodoma, P.O. Box 259, Dodoma, Tanzania; 2 Department of Nursing Management and Education, School of Nursing and Public Health, The University of Dodoma, P.O BOX 259, Dodoma, Tanzania

**Keywords:** MPDSR, Maternal death, Perinatal death

## Abstract

**Background:**

When used effectively, the Maternal and Perinatal Death Surveillance and Response (MPDSR) system can bring into reality a revolutionary victory in the fight against maternal and perinatal mortality from avoidable causes. This study aimed at determining the status of implementation of the system among health facilities in the Morogoro Region.

**Method:**

This study was conducted among 38 health facilities from three districts of the Morogoro region, Tanzania, from April 27, 2020, to May 29, 2020. Quantitative data were collected through document review for MPDSR implementation status. The outcome was determined by using a unique scoring sheet with a total of 30 points. Facilities that scored less than 11 points were considered to be in the pre-implementation phase, those scored 11 to 17 were considered in the implementation phase, and those scored 18 to 30 were considered to be in the institutionalization phase.

**Results:**

The majority 20(53 %) of health facilities were in the pre-implementation phase, only 15(40 %) of assessed health facilities were in the implementation phase, and few 3(8 %) of health facilities were in institutionalization phase. There was a strong evidence that MPDSR implementation was more advanced in urban compared to rural health facilities (Fisher’s test = 6.158, *p *= 0.049), hospitals compared to health centers (Fisher’s test =14.609, *p *<0.001) and private and faith-based organization than public facilities (Fisher’s test, 15.897 = *p *= 0.002).

**Conclusions:**

The study revealed that health facilities in Morogoro Region have not adequately implemented the MPDSR system. The majority of health facilities in rural settings and owned by the government showed poor MPDSR implementation and hence called for immediate action to rectify the situation. Strengthen MPDSR implementation, health facilities should be encouraged to adhere to the available MPDSR guidelines in the process of death reviews. Transparent systems should also be established to ensure thorough tracking and follow-up of recommendations evolving from MPDSR reviews. Health facilities should also consider integrating MPDSR to other quality improvement teams to maximize its efficiency.

## Background

It is estimated that 295 000 maternal death occurred in 2017 worldwide [1]. The burden of maternal deaths is in developing countries where maternal deaths are 40 times higher than in Europe and 60 times higher than in Australia and New Zealand [1]. Sub-Saharan Africa and South Asia have the highest maternal mortality, contributing up to 86 % maternal mortality globally [1]. Tanzania is among the sub-Saharan countries with the highest maternal mortality. The maternal mortality ratio in Tanzania is as high as 556 maternal death in every 100,000 live births [2].

It has been reported that the most leading causes of maternal deaths are haemorrhage (severe bleeding mainly after childbirth), infections (usually after childbirth), high blood pressure during pregnancy (pre-eclampsia and eclampsia), and unsafe abortion [3]. These five causes alone contributed to up to 80 % of maternal deaths in 2017 globally [3]. The remaining 20 % were caused by other causes such as when pregnancy was aggravated by other diseases like malaria, HIV, and the like [3]. These causes are preventable if skilled attendants attend pregnant women during pregnancy, childbirth, and the postnatal period.

Similar to maternal mortality, perinatal mortality rate is unacceptably high worldwide. The survival of a foetus and new-borns depends on the health status of their mothers. Perinatal mortality can be defined as foetal death at or after 28 weeks of gestation (stillbirth) or neonatal death within seven days of life (early neonatal mortality) [4]. Sub-Saharan Africa region is leading with a perinatal mortality rate of 34 perinatal deaths per every1000 live births. Tanzania is among the Sub-Saharan African country with the highest perinatal mortality, 39 perinatal deaths per every 1000 live birth [5].

The WHO target to reduce maternal and perinatal mortality is in the global 2030 agenda of the United Nations Development Program through the sustainable development goals number 3.1 and 3.2, which aims at decreasing maternal mortality ratio to less than seventy out of one hundred thousand live births. Furthermore, to end preventable deaths of neonates to less than twelve per a thousand live births [6]. The goals, which are set to be achieved by 2030, have been adopted by all countries under United Nations [7], including Tanzania.

Maternal and Perinatal Death Surveillance and Response (MPDSR) is a system of audit or review of maternal and perinatal deaths to improve health services and, hence, improve health services and prevent future maternal and perinatal deaths [8]. The system was issued by the World Health Organization in 2013 to help developing countries improve maternal health [8]. The primary purpose of the system is to reduce the ongoing high numbers of maternal deaths and perinatal deaths from avertable causes [9]. Since its introduction, MPDSR has become an important tool to help countries achieve the global targets in maternal health, and many countries have now managed to develop their own deaths surveillance and response systems [8].

Tanzania started to review maternal and perinatal deaths in 1984 with limited abilities in identifying the gaps [10]. In 2006 the health ministry launched the first death review guideline, but it focused only on maternal deaths. The implementation of the guideline was not successful due to a weak program monitoring system and inadequate competence to analyse the problems that caused deaths [10]. For the deaths which were ever recorded, it was done so without an organized system which was likely to lead to misclassifications and underreporting of causes of deaths [11]. Therefore a more effective system was needed in Tanzania, which would generate valid data by ensuring that all pregnancy-related deaths of women and new-borns or unborn babies around the time of delivery are well-reviewed by experts in the field [12].

MPDSR has two components; the surveillance component, which is a form of constant tracking of deaths of maternal and perinatal origin and connecting to the health information system and upgrading quality process from facility or community levels to national levels. The second part is the response component, which involves identifying problems that caused deaths, making action plans, implementing the action plans, and following up on agreed action plans [13]. MPDSR also offers information on current practices and provides suggestions and actions to be taken to abolish preventable maternal and perinatal deaths [12]. Therefore, when the system is implemented appropriately, it allows a complete understanding of the chain of events associated with maternal and perinatal death. recognizes the fatal problems in the whole process of caring for the patient from societal level to admission until the time of death and then suggests the best line of action strengthen health services so that similar scenario would not claim another innocent life [10].

Every health facility in Tanzania that provides reproductive and child health services,, including assisting childbirth, implements the MPDSR system [14]. The standard procedures for MPDSR require that every maternal death and perinatal death occurring either at the facility or in the community be reported to the regional level through respective councils, followed by a detailed review of the cause of death [10]. At the facility level, the meetings must be organized and led by senior facility leaders. They must involve critical cadres of the facility where death has occurred, such as clinicians, nurses, anaesthetists, laboratory personnel, and pharmaceutical personnel, including representatives from the council level [10]. During the case review, it is emphasized that neither blames nor identification should be made to staff who attended the deceased instead, the meeting should be focused on finding the gaps during care of the patient before death, this part ensures that the health worker builds a good attitude towards the system.

Despite the MPDSR implementation system in the country for years, the trend of maternal and neonatal mortalities is not promising. The majority of these deaths occur in rural settings of the country, which makes one wonders about the impact of the MPDSR system in addressing the challenge of maternal services provided in these settings. Little was known on the implementation status of the MPDSR system in Tanzania, specifically in Morogoro Region. Therefore, the study aimed at describing the implementation status of the MPDSR system in health facilities of the Morogoro Region.

## Methods

### Study setting

The study was conducted in the Morogoro region, which is located in Eastern Tanzania. Morogoro is the second-largest region in the country [15]. This administrative region is bordered by the coast and Lindi regions in the East, Manyara and Tanga regions in the north, Dodoma and Iringa regions in the west, and Ruvuma region in the south [15]. Morogoro Region has six districts which are Morogoro, Gairo, Mvomero, Kilosa, Kilombero and Ulanga [15].

Morogoro region was chosen to be the study location because of its large number of health facilities and high maternal and perinatal mortality rate. No study related to MPDSR had been done in the region before the Morogoro region was chosen to be the location of the study because of its large number of health facilities, high maternal and perinatal mortality rate. Health services in the Morogoro region are provided mainly by the government and faith-based organizations. With 552 operating health facilities (15 hospitals, 52 health centres, and 378 dispensaries), the Morogoro region is among the top five regions with a high volume of health facilities in Tanzania [16]. Health services in the Morogoro region are provided mainly by the government and faith-based organizations. With 552 operating health facilities (15 hospitals, 52 health centres, and 378 dispensaries), the Morogoro region is among the top five regions with a high volume of health facilities in Tanzania [16].

### Study design

An analytical cross-sectional study design using a quantitative approach was used to assess the status of MPDSR implementation in health facilities. Data were collected by using documentary review and observation methods with a guiding checklist.

### Inclusion criteria

A facility that was registered to deliver health services as a hospital or a health centre. Dispensaries were not included in the assessment because of the level of maternal and child services provided at this level. In Morogoro and Tanzania at large, dispensaries provide essential obstetric care; if a complication is diagnosed, the mother is referred to a second level or third level depending on the distance to a nearby referral point. Health facilities and hospitals provide both primary and comprehensive obstetric care, and in these levels, most maternal and perinatal deaths occur.

### Exclusion criteria

Hospitals and health centres that did not offer reproductive, maternal and child health services were excluded.

### Sample size calculation

The sample size was estimated by using the formula of cross-sectional study for finite population [17], as shown in Eq. 1,

1 $$n=\frac{z^2P\left[1-P\right]}{{Ne}^2+z^2P\lfloor1-P\rfloor}$$

**Where**,

- n = desired sample size,
- z = critical value for 95 % confidence level which is 1.96,
- e = desired margin error which is 0.05,
- N = the size of the target population, which was 62 and,
- P = proportion of health facilities that showed evidence of MPDSR implementation from a study conducted in Lake zone, Tanzania = 93.8 % [10].

Then, sample size n was obtained from the following calculation;

$$n= \frac{{1.96}^{2}*0.938\left[1-0.938\right]}{{62*0.05}^{2}+{1.96}^{2}*0.938\lfloor1-0.938\rfloor}=38$$

### Sampling technique

A multistage sampling technique was applied during facility selection. A purposive selection of three councils from the Morogoro region was made based on the high number of health facilities. The selected facilities were Morogoro MC, Mvomero DC, and Kilosa DC. In each council, all hospitals were conveniently selected. Therefore, 11 hospitals were included in the study (Morogoro MC = 3, Mvomero DC = 4, and Kilosa DC = 3). The remaining 27 (after subtracting 11 facilities from 38) facilities were health centres that were stratified by the council to obtain the adequate representation of each council. Since the number of health centers from the three councils was 14, 8, and 7 respectively, each council’s representative number of health facilities was calculated.

$$\mathrm{Total}\ \mathrm{number}\ \mathrm{of}\ \mathrm{health}\ \mathrm{centers}=14+7+8=29,$$

Then,

$$\mathrm{Number}\ \mathrm{of}\ \mathrm{health}\ \mathrm{centers}\ \mathrm{from}\ \mathrm{Morogoro}\ \mathrm{MC}=27\ast \frac{14}{29}=14$$

$$\mathrm{Number}\kern0.17em \mathrm{of}\kern0.17em \mathrm{health}\kern0.17em \mathrm{centers}\kern0.17em \mathrm{from}\kern0.17em \mathrm{Mvomero}\;\mathrm{DC}=27\ast \frac{8}{29}=8$$

$$\mathrm{Number}\kern0.17em \mathrm{of}\kern0.17em \mathrm{health}\kern0.17em \mathrm{centers}\kern0.17em \mathrm{from}\kern0.17em \mathrm{Kilosa}\;\mathrm{DC}=27\ast \frac{7}{29}=7$$

Finally, the required number of health centers was selected from each council by a simple random technique by replacement using the lottery method.

### Data collection procedure

Data were collected through document review and observation methods. A principal researcher with one assistant visited the selected health facilities and asked the facility in charge or any other persons appointed by the in-charge to provide them with necessary MPDSR reports and documents. Labour wards of respective facilities were also visited for observation purposes with regards to MPDSR practice. Data were collected during the outbreak of the Covid-19 pandemic. The pandemic did not affect the completeness of the data expected to be collected but instead affected the duration of data collection. The study proposed collecting data for only four weeks, but the actual time for data collection was extended to six weeks. The extension was due to extended procedures for data collection and the availability of host health workers to assist in data acquisition.

### Variable measurement

MPDSR implementation status was measured using a special scoring scale modified from a tool used in a previous study in Lake Zone, Tanzania [10]. The tool had a maximum of 30 points. Data from both the documentary review and observation were used to assign scores to the facility. A score of less than 11 was termed as MPDSR pre-implementation phase, A score of 11-17 was termed as MPDSR implementation status, and the score of 18 to 30 was termed as MPDSR institutionalization. In the pre-implementation phase, the facility has created awareness on the MPDSR system, adopted the system, and took ownership of the system. In the Implementation phase, the facility has created awareness of the MPDSR system, adopted the system, took ownership of the system, showed the MPDSR system, adopted the system, took ownership of the system, and showed evidence of MPDSR practice. In the institutionalization phase, the health facility has created awareness on MPDSR system, adopted the system, taking ownership of the system, showed evidence of MPDSR practice, showed evidence of routine integration lesson learnt from review and has sustainable MPDSR practice.

### Data processing and analysis

Data were entered into SPSS software for cleaning and analysis. Descriptive statistics, which were mean, proportions, frequency distribution, and Chi-square, were used to measure the MPDSR implementation status in facilities.

## Results

### Facility characteristics

Out of 38 health facilities enrolled in the study, 11 (29 %) were hospitals, while 27 (71 %) were health centers. Ten (26 %) health facilities were located in urban and 25 (66 %) were owned by the government. See Table 1.

**Table 1:** Facility characteristics (*n* = 38)

Variable	Frequency (n)	Percentage (%)
Level		
Hospital	11	29
Health Centre	27	71
Location		
Urban	10	26
Rural	28	74
Ownership		
Public	25	66
Private	13	34

### MPDRS implementation assessment

Table 2 shows that 27 (71 %) health facilities had no guidelines regarding MPDSR, although 37 (97 %) had data collection forms in place. Death review meetings were shown to be held at a stated interval in 1 (3 %) facility. Of all assessed facilities none of them had MPDSR data trends displayed or shared, and none could show evidence of change based on recommendations arising from death review findings. All 38 (100 %) health facilities had particular persons who take a specific effort in promoting death reviews meeting as a coordinator. Furthermore, the MPDSR coordinators from all 38 (100 %) health facilities had other responsibilities. Facilities in charge were shown to chairs the MPDSR meeting in 21 (55 %) health facilities. No facility had evidence of staff receiving MPDSR training for the past one here before the study.

**Table 2:** MPDSR tools and protocols (*n* = 38)

Item	YES n (%)	NO n (%)
There are written policies, guidelines or protocols regarding the practice of MPDSR	11 (29)	27 (71)
Data collection forms are available	37 (97)	1 (3)
Tools include causes of deaths	35 (92)	3 (8)
Tools include modifiable factors for the cause of death	35 (92)	3 (8)
Tools include a place to follow up on actions taken	3 (8)	35 (92)
Attendance is mandatory	20 (53)	18 (47)
Death review meetings is held at the stated interval	1 (3)	37 (97)
Data trends are displayed or shared	0 (0)	38 (100)
Evidence of change based on recommendation arising from death review findings	0 (0)	38 (100)
Unique persons who take a specific effort in promoting death reviews, including management, professionals, driving forces	38 (100)	0 (0)
The coordinator(s) have other responsibilities (e.g. information officer. I.Q.I. focal point, etc.)	38 (100)	0 (0)
Clear leader(s) involved in establishing and championing death reviews	36 (95)	2 (5)
Has anyone in facility or district leadership signed a commitment or undertaken an agreement that s/he would ensure that MPDSR is implemented in the facility?	0 (0)	38 (100)
The facility in charge chairs the MPDSR meeting	21 (55)	17 (45)
Evidence that staff have received MPDSR training in the past year	0 (0)	38 (100)

### MPDSR implementation status

**Fig. 1:**
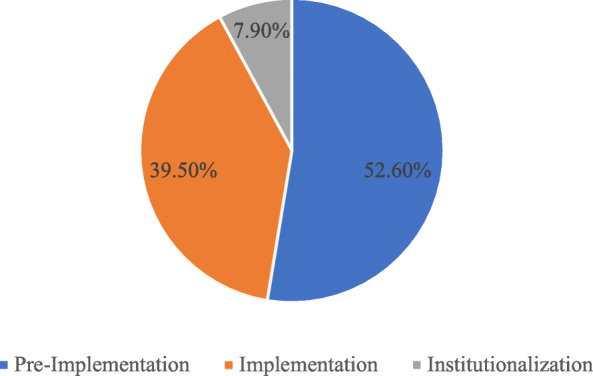
The MPDSR implementation status

The mean score of implementation status was 10.5 points, the maximum score being 20 points while the minimum score being 5 points. For the implementation status, 20(53 %) were in the pre-implementation phase, 15(40 %) were in the implementation phase, and 3(8 %) were in the institutionalization phase, as shown in Fig. 1 below.

**Fig. 2:**
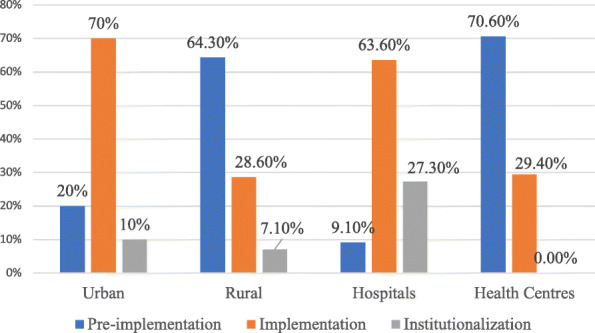
MPDSR implementation status according to location and facility type

The majority of health facilities in rural settings had pre-implementation status. Regardless of the location of health facilities, health centres, in general, had pre-implementation status, see Fig. 2.

### The relationship between facility characteristics and status of MPDSR implementation

Variables that showed a significant relationship with the MPDSR implementation were the place of location of health facility (urban or rural), Fisher’s test = 6.158, *p *= 0.049, level of health facility (hospital or health center), Fisher’s test =14.609, *p *<0.001 and Ownership of the facility Fisher’s test, 15.897 = *p *=0.002, see Table 3.

**Table 3:** The relationship between facility characteristics and status of MPDSR implementation

Variables	Pre implementation n(%)	Implementation n(%)	Institutionalization n(%)	Fisher’s test	*P*-value
**Council**				7.415	0.129
Morogoro Municipal	2(20)	7(70 %)	1(10)		
Mvomero DC	11(73)	3(20)	1(7)		
Kilosa DC	7(54)	5(39)	1(8)		
**Place of location**				6.158	0.049
Urban	2(20)	7(70)	1(10)		
Rural	18(64)	8(29)	2(7)		
**Level of health facility**				14.609	<0.001
Hospitals	1(9)	7(64)	3(27)		
Health centers	19(71)	8(29)	0(0)		
**Ownership of a facility**				15.897	0.002
Public	18(72)	4(16)	3(12)		
FBO	0(0)	3(100)	0(0)		
Private	2(20)	8(80)	0(0)		

## Discussion

The implementation of the MPDSR system is generally not satisfactory in most health facilities in the Morogoro Region. It was found that more than half of the health facilities involved in the study had MPDSR pre-implementation phase. Thus, the facilities have created awareness on the MPDSR system, adopted the system, and took ownership of the system, still these health facilities, cannot show evidence that the system is practised. This is an alarming situation because it deviates from the ultimate aim of the MPDSR system, which is to improve the quality of maternal services provision through review of maternal and perinatal deaths and use the report to improve the practice. Through maternal or perinatal death review, there are lessons learnt for future practice. A well-directed effort is highly needed to facilitate the implementation of the MPDSR system in Morogoro.

The study also found that 40 % of health facilities were in the MPDSR implementation phase. In this phase, the facilities have created awareness of the MPDSR system, adopted the system, took ownership of the system, and showed evidence of MPDSR practice. At this level, the facility lacks the evidence that the data obtained from the review are used to improve the practice. Different findings were reported by a similar study done in four sub-Saharan countries where 44 % of studied health facilities could demonstrate evidence that reduction in maternal and perinatal mortalities was due to MPDSR implementation [18]. The possible reason could be the differences in the coverage in the two studies. This observation casts light on the need to explore further the implementation fidelity of the system. Schmiegelow and others [19], reported similar findings, also a similar study conducted in Tanzania found that not all hospitals had a functional Maternal and perinatal audit system in place, concluding that the MPDSR system is not implemented following the expectations [20].

Contrary to the findings, a previous similar study done in Kagera and Mara reported that the low level of MPDSR implementation status was due to differences in information collection and quality of data among facilities; this study found that more than ninety per cent of facilities had similar tools of documenting deaths. Although all health facilities had a formal system of reviewing deaths and had a person who coordinates the process of death reviews, meetings were not done at regular intervals to the most health facilities contrarily to the requirement [10]. The lack of regular meetings could have slowed down the MPDSR implementation process.

Further in this study, it was found that there was a significant relationship between facility characteristics and the MPDSR implementation status, such as level of health facility, ownership of the facility and setting the facility is located. Majority of health facilities located in the rural setting had MPDSR pre-implementation status. This means that no evidence that the MPDSR system is practiced. It is the same setting in the country with the highest maternal and neonatal mortalities [21]. This is alarming, and a deliberate effort is needed to empower the facilities to implement the MPDSR system. The council, health management team are the focal technical team to facilitate the implementation of the MPDSR system. This observation shows the influence of management in health systems, supporting the need to review health policies that will help improve health services [22].

Similarly, the majority of health centers had MPDSR pre-implementation status. This could be due to the workforce in this level have inadequate training compare to the workforce working in the hospitals. There is the necessity of regular capacity training in the health centres workforce to raise awareness of MPDSR implementation. Furthermore, the majority of public-owned health facilities had pre-implementations status. This could be due to a crisis of both human and non-human resources for health.

Nevertheless, a deliberate effort is needed to improve the MPDSR implementation system. An empowered health workforce on the implementation of the system will facilitate the implementation of the system. The feedback obtained from the review can facilitate -effective cost distribution of the available resources.

In this study, it was also found that all assessed facilities had MPDSR coordinators who had other responsibilities in contrast to the MPDSR guideline [10]. This could be because of staff shortage demonstrated in the study by (MCSP 2018). Moreover, it could explain insufficient response to MPDSR implementation despite staff commitment that has already been observed [20].

The study also demonstrated the lack of management planning for effective MPDSR implementation. This evidenced by the finding that none of the facilities MPDSR data trends is displayed or shared. None of the facilities had documented evidence of change due to MPDSR systems. None of them had a plan in place to ensure all staff receives MPDSR training. And most of the facilities did not conduct review meetings at a regular interval which could all influence the status of MPDSR implementation.

The study was not without limitations; it was a descriptive study that aimed at establishing the MPSDR implementation status in Morogoro Region. The findings from this study laid a foundation for further studies that will inform why some facilities perform better than others and facilitates the development of innovative strategies that will improve MPDSR implementation status. The MPDSR implementation status was assessed using 30 items checklist, the criteria for categorising them into the three categories based on the previous study which was done arbitrary, this could have affected the implementation status reported. The study recommends the development of a standard tool of assessing MPDSR implementation status. Also, the study did not include the dispensaries but rather the health centers (first referral point) and hospital (the second referral point), majority of maternal and neonatal mortalities occur in these referral points. The findings from this study provide foundation for a bigger study which will include all facilities. Furthermore, the study was conducted during the outbreak of the Covid-19 Pandemic, which could have affected the data collection. The impact of the Covid-19 Pandemic was minimized by adding more time for data collection.

## Conclusions

The study revealed that health facilities in Morogoro Region have not adequately implemented the MPDSR system. strengthen MPDSR implementation, health facilities should be encouraged to adhere to the available MPDSR guidelines in the process of death reviews. Transparent systems should also be established to ensure thorough tracking and follow-up of recommendations evolving from MPDSR reviews. Health facilities should also consider integrating MPDSR to other quality improvement teams to maximize its efficiency.

## Data Availability

The data and material used in the current study are available from the corresponding authors upon request.

## Abbreviations

AOR: Adjusted odds ratio
CEmOC: Comprehensive emergency obstetric care
CI: Confidence interval
DC: District council
DPG: Development Partner Group
MC: Morogoro Municipal
MCSP: Maternal child survival partnership
MoHCDGEC: Ministry of Health Community Development Gender Elder and Children
MPDSR: Maternal and perinatal deaths surveillance and response
OPD: Outpatient Department
OR: Odds ratio
SMI: Safe mother initiative
SPSS: Statistical package for social sciences
